# Supplementary material for: Uncertainty and reward histories have distinct effects on decisions after wins and losses
Source: bioRxiv. 2025 Aug 19:2025.08.14.670176. Preprint. [Version 1] doi: 10.1101/2025.08.14.670176 (PMC12393270; doi:10.1101/2025.08.14.670176)
Supplement: 1 [file NIHPP2025.08.14.670176v1-supplement-1.pdf]

bioRxiv preprint doi: https://doi.org/10.1101/2025.08.14.670176; this version posted August 19, 2025. The copyright holder for this preprint (which was not certified by peer review) is the author/funder, who has granted bioRxiv a license to display the preprint in perpetuity. It is made available under aCC-BY 4.0 International license.

Supplementary Materials

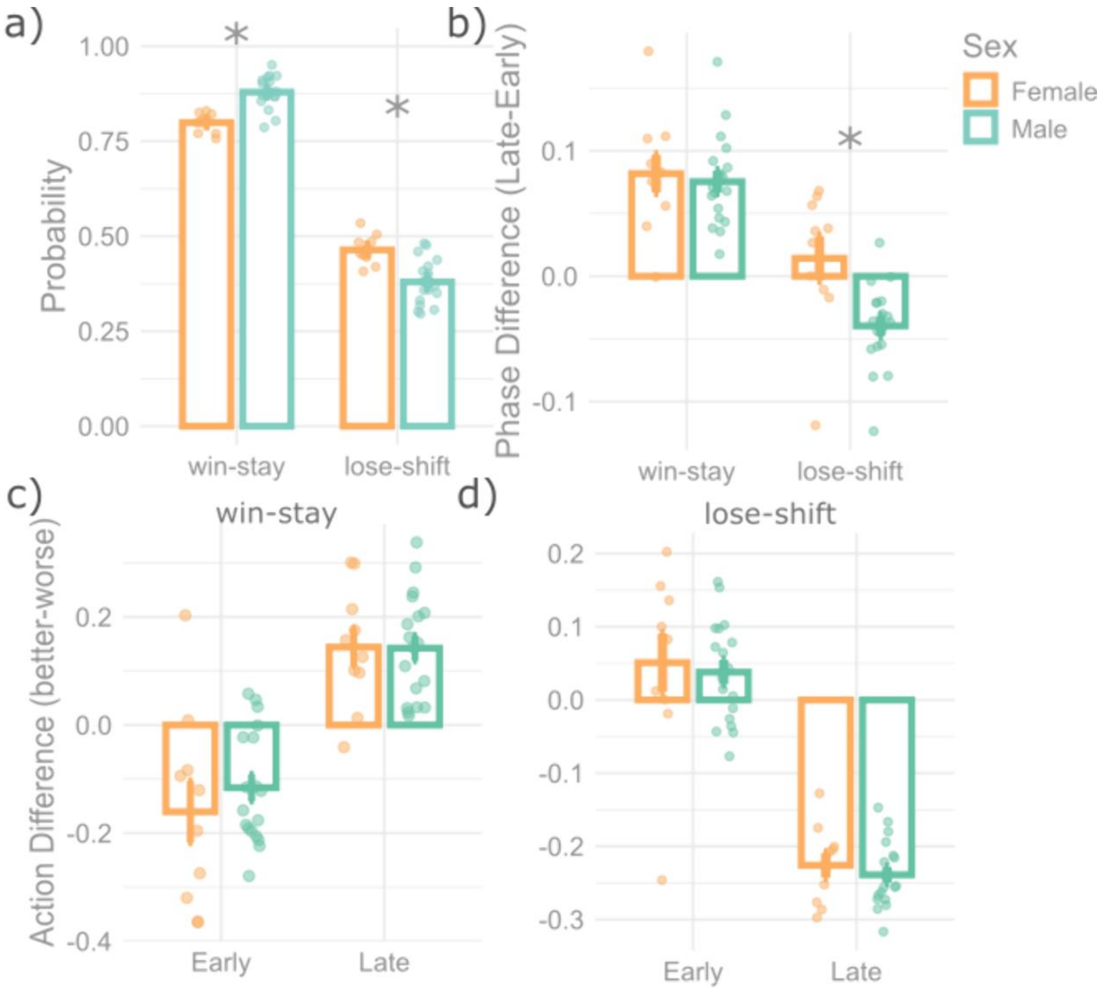

Figure S1. *Asymmetrical learning strategies in the probabilistic reversal learning task.* a) rats had a higher WS probability compared to the LS probability in the PRL task (main effect; F(1,52)=1076.6; p = 2.2e-16). There was an interaction between the WSLS factor and sex (F(1,52) = 41.83, p = 3.5e-08). Subsequent t-tests revealed that, females had a lower WS probability, but a higher LS probability, compared to males. **b)** asymmetry emerges for WS and LS behaviors (main effect of WSLS factor; F(1,26) = 66.55, p = 1.2e-08), where both males and females WS more in the late phase of the block, compared to the early phase. However, only males developed the strategy of also reducing their LS probability in the late phase of the block (p = 0.002, Cohen's d = 1.34). **c-d)** There were differences in WS and LS probabilities between phases (interaction between WSLS type and phase type; F(1,104) = 198.86, p = 2.2e-16). Subsequent t-tests revealed a significant difference in early versus late phase WS (p < 1e-04, Cohen's d = 2.80) and LS probabilities (p < 1e-04, Cohen's d = 2.76). In the late phase, rats were more likely to WS if that win was from the better action, compared to the worse action. Conversely, rats were less likely to LS if that loss was from this better action, compared to the worse action, which emerged in the late phase of the block.

bioRxiv preprint doi: https://doi.org/10.1101/2025.08.14.670176; this version posted August 19, 2025. The copyright holder for this preprint (which was not certified by peer review) is the author/funder, who has granted bioRxiv a license to display the preprint in perpetuity. It is made available under aCC-BY 4.0 International license.

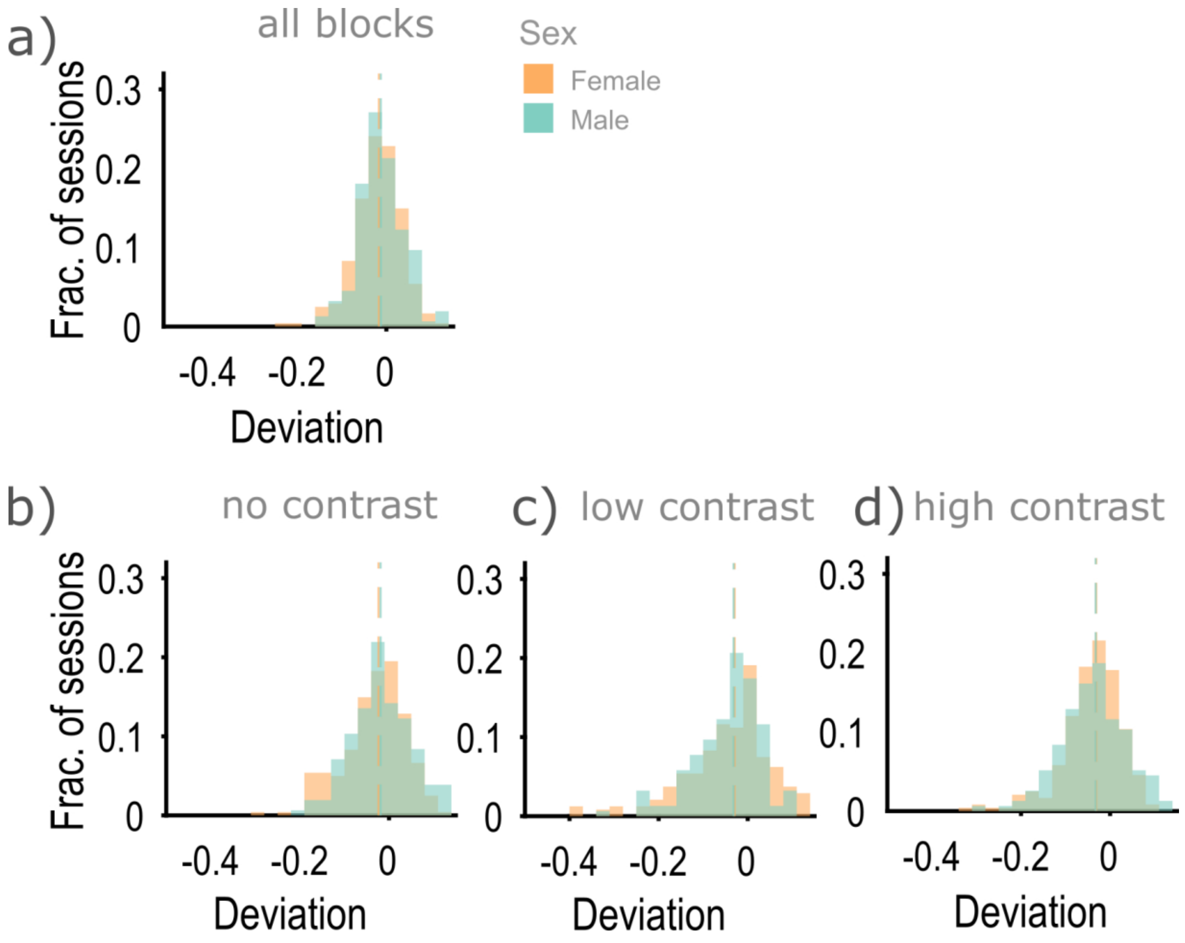

Figure S2. *Males and females deviate at similar levels from the matching behaviors*. **a)** Both sex groups have a higher matching score (i.e., a higher deviation of choice probability from the reward probability based on the choice). However, in across all blocks **(a),** and within the three block types **(b-d)**, there were no sex differences, indicating that males and females make similar choices based on their local reward outcomes.
